# Long-Term Effects of Radiation Therapy on Cerebral Microvessel Proteome: A Six Month Post-Exposure Analysis

**DOI:** 10.1021/acsomega.5c09726

**Published:** 2025-10-26

**Authors:** Vikram Subramanian, Denise Juhr, Piero Giansanti, Isabella M. Grumbach

**Affiliations:** 1 Abboud Cardiovascular Research Center, Department of Internal Medicine, Carver College of Medicine, 21710 University of Iowa, Iowa City, Iowa 52242, United States; 2 Bavarian Center for Biomolecular Mass Spectrometry at Klinikum rechts der Isar (BayBioMS@MRI), Technical University of Munich, 81675 Munich, Germany; 3 Free Radical and Radiation Biology Program, Department of Radiation Oncology, Carver College of Medicine, 21710 University of Iowa, Iowa City, Iowa 52242, United States; 4 Iowa City VA Healthcare System, Iowa City, Iowa 52246, United States

## Abstract

Radiation therapy (RT) treats primary and metastatic brain tumors, with about one million Americans surviving beyond six months post-treatment. However, up to 90% of these survivors develop RT-induced cognitive impairment. Emerging evidence links cognitive decline to RT-induced endothelial dysfunction in brain microvessels, yet *in vivo* studies remain limited. Investigating the molecular and cellular pathways connecting RT, endothelial injury, and cognitive impairment is vital for developing targeted interventions. We performed quantitative proteomic analysis of cerebral microvessels from five control and five irradiated mice (12 Gy) 6 months post-RT. Bioinformatics tools, including gene ontology (GO) enrichment, Mitocarta analysis, Ingenuity Pathway Analysis (IPA), and iPathwayGuide, identified affected pathways. Findings were validated by Western blotting. RT significantly altered 414 proteins, with 157 upregulated and 257 downregulated. GO analysis indicated metabolic pathway disruptions, and Mitocarta analysis revealed a significant presence of mitochondrial proteins among the dysregulated proteins. IPA identified 76 enriched canonical pathways, 34 transcription regulators, 6 nuclear receptors, and 5 growth factors involved in RT-induced damage responses. IPA predicted mitochondrial dysfunction in the irradiated group, confirmed by Western blotting. Significant proteomic changes in cerebral microvessels suggest RT-induced metabolic dysfunction in cerebral microvasculature, including oxidative phosphorylation, the TCA cycle, and glycolysis.

## Graphical abstract

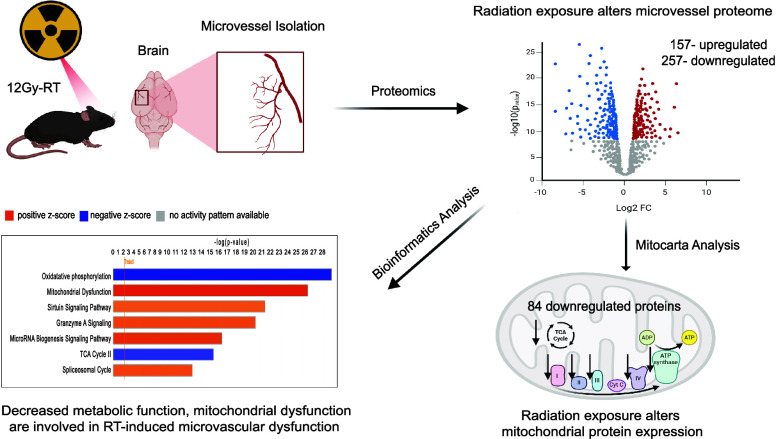

## Introduction

Microvascular injury is a hallmark of late radiation damage across various organs depending on the radiation field. It is believed to promote radiation-induced renal dysfunction, skin fibrosis and heart failure.^1–5^ One area where the effects of radiation on normal tissue are particularly apparent is injury after treatment of brain tumors.^6–8^ In fact, up to 90% of patients experience cognitive function decline within three to six months after radiation.^9–15^ Vascular damage, neuroinflammation and neuronal injury have been proposed as mechanisms driving cognitive decline.^16^ Microvascular injury can compromise the blood-brain barrier (BBB) breakdown, leading to inflammatory cell infiltration and inflammation.^17^ It can also result in apoptosis and senescence in vascular wall cells, potentially causing vascular rarefaction, reduced perfusion, and ischemia.^18^ While there is consensus on a role of microvascular injury, the exact mechanisms and extent of microvascular injury’s role in cognitive decline remains poorly understood. Previous studies have used proteomic approaches to characterize the effect of radiation exposure on mouse whole brain proteome,^19^ hippocampal proteome,^20^ vascular endothelial membrane proteome,^21^ and microglial signaling pathways.^22^ However, to date, no study has investigated the long-term impact of radiation on the brain microvasculature using label free quantitative proteomics approach. Such analysis is critical to understand radiation-induced microvascular injury and its role in cognitive decline.

To address this gap, we aimed to uncover changes in the microvascular proteome 6 months after RT. We chose to conduct our study using 12-month-old mice, approximately equivalent to 60-year-old humans, which is close to the reported median age of patients treated with RT for brain tumors.^23^ Of note, there is age-dependent variation in brain radiation injury, with the highest pathology occurring in the very young and elderly.^16,24^ We opted for a six-month follow-up to focus on chronic changes from radiation injury. Previous work, including our own, has suggested that various pathways, such as mitochondrial injury, altered vascular transport, and changes in cell adhesion and tight junctions, may play a role in the long term effects of radiation.^25–29^ To gain an unbiased view on long-term effects of radiation exposure on the cerebral microvessel proteome, we conducted bioinformatics analyses using Ingenuity Pathway Analysis (IPA) and iPathwayGuide to identify significantly affected canonical pathways that may contribute to cerebral microvasculature dysfunction. Additionally, we performed immunoblotting to validate our proteomics findings and bioinformatics results.

## Materials and Methods

### Animals Housing and Irradiation Procedure

One-year-old male C57BL/6J mice were obtained from the Jackson Laboratory. The mice were randomly assigned to the treatment (RT) or the control group, each with five animals. They were housed in temperature-controlled rooms with a 12-h light/dark cycle, provided standard rodent chow, and had water access *ad libitum*. To avoid gender-related confounding factors, only male mice were used. The control group mice (n = 5) underwent sham irradiation, anesthetized with ketamine and xylazine, placed in the radiation chamber without exposure. Mice were randomly selected for irradiation versus sham treatment. The irradiated group (n = 5) received a 12 Gy X-ray dose to the whole brain using the XStrahl Small Animal Radiation Research Platform, which uses a 60 kVp beam of 0.2 mm Al quality for Cone Beam CT and a 220 kVp 0.63 mm Cu quality beam for treatment. The 12 Gy dose corresponds to 2 Gy fractions of a 40 Gy total, commonly used for RT in patients with five or more brain metastasis. Both groups were euthanized six months postirradiation for analysis. No mice were excluded from the analysis. All experimental procedures were carried out by following Guide for the Care and Use of Laboratory Animals and approved by the Institutional Animal Care and Use Committees of the University of Iowa and the Iowa City VA Health Care System. This study was conducted in accordance with the ARRIVE guidelines 2.0 for reporting animal research to ensure transparency, rigor and reproducibility.

### Isolation of Cerebral Microvessels and Preparation of Protein Samples

Mice were euthanized using 100% CO2 inhalation, followed by cervical dislocation. Brains from both control and irradiated groups were surgically removed, rinsed with cold Dulbecco’s phosphate-buffered saline (DPBS) (Gibco #2430024), snap-frozen in liquid nitrogen, and stored at – 80 °C. Cerebral microvessels were isolated following Lee et al.^30^ Briefly, brain tissue was homogenized with a loose-fit Dounce grinder (Sigma-Aldrich #D9063) in MCDB 131 medium (Thermo Fisher Scientific #10372019) and centrifuged at 2,000 g for 15 min at 4 °C. The pellet was resuspended in a 15% (wt./vol) 70-kDa dextran solution (Sigma-Aldrich #31390) and centrifuged at 7,000 g for 15 min at 4 °C. The top layer containing myelin and parenchymal cells was discarded. Microvessels were collected using a 40-μm cell strainer (Corning #352340), rinsed with cold DPBS, and transferred into MCDB 131 medium containing 0.5% (wt./vol) BSA (Millipore Sigma #126609). The suspension was centrifuged at 16,100 g for 30 min at 4 °C to pellet the microvessels. Pellets were stored at – 80 °C for analysis.

For protein extraction, the isolated microvessel pellets were mixed with 130 μL of RIPA buffer (Fisher Scientific #R0278) containing protease (Pierce #A32963) and phosphatase inhibitor cocktails (Pierce #A32957). The mixture was vortexed three times for 30 s each (Fisher Scientific #0215370), then agitated for 30 min at 15–20 °C. Samples were vortexed again and heated at 95 °C for 10 min. To further break down the tissue, the samples were lysed using a Covaris E220 focused ultrasonicator (Covaris #500239) with following parameters. The instrument parameters for shearing were as follows: water level set point 10, water temperature 6 °C, peak incident power 175 W, duty factor 10%, cycles per burst 200, and duration 300 s. Homogenized lysates were transferred to Eppendorf tubes and centrifuged at 16,100 g (Eppendorf #5415R) for 30 min. The supernatant was collected and stored at – 80 °C. Total protein concentration was measured using a BCA protein assay kit (Pierce #23225). Finally, 30 μg of protein from each sample was used for quantitative proteomics analysis. Samples were labeled with a numeric identifier that did not include information about irradiation or sham treatment and sent for proteomic analysis.

### Protein Digestion

Protein digestion was performed using the SP3 method.^31^ For each sample, 30 μg of protein in 150 μL of lysis buffer was incubated with 10 μL of SP3 beads (a 1:1 mixture of Sera-Mag Speed Beads A and B, Thermo Scientific). Pure acetonitrile (ACN, VWR Chemicals) was added to a final concentration of 70% (v/v). The samples were incubated mixed at 800 rpm for 18 min, then placed on a magnet rack for 3 min to immobilize the SP3 beads. The supernatant was discarded, and the SP3 beads were washed three times with 1 mL of 80% ethanol/water (v/v) and once with 800 μL of ACN. Bound proteins were reduced with 100 μL of 10 mM 1,4-dithiothreitol (DTT, Sigma) in 50 mM ammonium bicarbonate (Sigma), pH 8.0, and incubated at 37 °C with shaking at 800 rpm for 1 h. Proteins were then alkylated with 55 mM 2-chloroacetamide (CAA, Sigma) for 1 h at 37 °C in the dark. Finally, 1 μg of trypsin (Thermo Scientific) was added to each sample, and they were incubated overnight at 37 °C with shaking at 800 rpm. Digests were acidified with formic acid (FA, Carlo Erba) to a final concentration of 1% (v/v), dried in vacuo, and stored at – 80 °C until use.

### Automated Offline Fractionation and LC-MS/MS Analysis

Peptides were resuspended in 110 μL of buffer A (25 mM ammonium formate, pH 10) and subjected to high pH reverse-phase fractionation using the AssayMAP Bravo platform and 5 mL RP-S cartridges (Agilent). Cartridges were primed with 150 μL each of isopropanol, acetonitrile (ACN), and buffer B (80% ACN in 10 mM ammonium formate, pH 10) at a 50 μL/min flow rate. They were then equilibrated with 100 μL of buffer A, and peptides were loaded at 5 μL/min, collecting the flow-through (FT). Peptides were eluted with 25 mM ammonium formate, pH 10, using increasing ACN concentrations (5%, 10%, 15%, 20%, 25%, 30%, 80%). The seven fractions were pooled into four final fractions, dried in vacuo, and stored at – 80 °C.

Nanoflow LC-MS/MS was performed using a Dionex Ultimate 3000 UHPLC+ system coupled to an Orbitrap Eclipse mass spectrometer (Thermo Fisher Scientific). Peptides were first delivered to a trap column (75 μm i.d. × 2 cm, packed in-house with 5 μm Reprosil C18 beads) and washed with 0.1% formic acid (FA) at 5 μL/min for 10 min, then transferred to an analytical column (75 μm i.d. × 45 cm, packed in-house with 3 μm Reprosil C18 beads) at 300 nL/min. Chromatographic separation used a linear gradient of solvent B (0.1% FA, 5% dimethyl sulfoxide (DMSO) in ACN) and solvent A (0.1% FA, 5% DMSO in water) over a 90 min total run time.

Full-scan MS spectra were recorded in the Orbitrap from 360 to 1,300 *m*/*z* at 60,000 resolutions, with an automatic gain control (AGC) target of 100% and a maximum injection time (maxIT) of 50 ms. The most intense precursors were isolated with a 1.3 *m*/*z* isolation window for high-energy collisional dissociation (HCD) fragmentation. Fragment ions were recorded in the Orbitrap at 15,000 resolutions, with a maxIT of 22 ms and an AGC target of 200%. Normalized collision energy (NCE) was set to 25%. Charge state screening selected precursors with charge states 2 to 6 for fragmentation, within a 2-s cycle time. Dynamic exclusion was set to 35 s.

### Identification and Quantitation of Peptides and Proteins

Raw mass spectrometry data were processed using MaxQuant (version 2.2.0.0) with its built-in search engine, Andromeda.^32^ Spectra were searched against the UniProtKB database for *Mus musculus* (UP000000589, 55,338 entries, downloaded October 2022). Enzyme specificity was set to trypsin, allowing up to two missed cleavages. The search included cysteine carbamidomethylation as a fixed modification, and protein N-term acetylation and methionine oxidation as variable modifications. Identifications were filtered to achieve a 1% false discovery rate (FDR) at protein and peptide levels. The ″match between runs″ and ″second peptide″ options were enabled. Label-Free Quantification (LFQ) was performed using the MaxLFQ algorithm.^33^ The mass spectrometry proteomics data have been deposited in the ProteomeXchange Consortium via the PRIDE repository^34^ with the data set identifier PXD058732 (Web site: http://www.ebi.ac.uk/pride, Username: reviewer_pxd058732@ebi.ac.uk, Password: RJJaEf7seviy).

### Proteomics Data Analysis and Pathway Enrichment Analysis

Protein identifications were filtered to exclude contaminants and reverse (decoy) hits. Label-free quantification (LFQ) intensity values were log_2_-transformed and normalized by median centering **(** Table S1 **)**. For downstream analysis, only proteins quantified in all biological replicates were retained **(n = 2,457,** Table S2 **)**. Differential protein expression was assessed using a Student’s *t* test with an S_0_ parameter of 0.1. To account for multiple testing, a permutation-based false discovery rate (FDR) correction was applied, with a significance threshold set at FDR < 0.05. This procedure is implemented in Perseus using the Benjamini-Hochberg method, where statistically significant proteins are flagged (+) rather than reporting explicit q-values. Proteins identified by at least two unique peptides, with a fold change <0.77 or >1.30 and meeting the corrected p-value threshold, were considered significantly differentially expressed (n = 414, Table S3)^35–37^ and these proteins were used for downstream analysis. Hierarchical cluster analysis of significantly dysregulated proteins based on LFQ intensity values was performed using SR plot.^38^ A volcano plot was generated using VolcaNoseR web tool^39^ including only proteins that met the adjusted p-value (q-value) threshold. As numerical q-values were not available from Perseus, the plot was created using unadjusted p-values for the proteins identified as significant, to illustrate differentially expressed proteins. To determine which proteins in our data set are likely derived from specific vascular cell types (endothelial cells, pericytes and astrocytes), we compared the full quantified proteomics data set (2,457 proteins) with a reference single-cell transcriptomic atlas of the mouse brain vasculature, based on single-cell RNA-seq gene expression data.^40^

Enrichment analysis of dysregulated proteins was conducted to assess their impact on biological processes, cellular locations, molecular functions, and KEGG pathways after radiation exposure, using HemI 2.0.^41^ Canonical pathway, upstream regulator and disease and biofunction analyses were performed with INGENUITY Pathway Analysis (IPA) software,^42^ applying a threshold of – log (p-value) > 1.3. Pathways with a z-score >2.0 were considered activated, while those with a z-score < – 2.0 were inhibited. Impact pathway analysis on differentially expressed proteins was conducted using iPathwayGuide^43^ with pathway annotations from the KEGG database and gene ontology annotations from the Gene Ontology Consortium database. A threshold of FDR-corrected p-value <0.05 was used to identify significantly affected pathways.

### Immunoblotting Analysis

Equal amounts of protein (5–10 μg) from control and irradiated groups were separated on NuPAGE 4% to 12% Bis-Tris gels (Life Technologies) and transferred to polyvinylidene difluoride (PVDF) membranes using the Mini-PROTEAN 3 blotting system (Bio-Rad). Membranes were stained with Ponceau S (Cell Signaling #59803S) to confirm protein transfer, washed in TBS with Tween (TBST), and blocked with 3% BSA (RPI Research #9048–46–8) or Starting Block Blocking Buffer (Thermo Fisher Scientific #37538) for 2 h. Membranes were incubated overnight at 4 °C with primary antibodies, including anti-NDUFB8 (Abcam, no. ab110413), anti-NDUFA11 (ABclonal, no. A16239), anti-NDUFA4 (ABclonal, no. A15693), anti-SDHA (Cell Signaling, no. 11998), anti-SDHB (Abcam, no. ab110413), anti-UQCRC2 (Aviva systems biology, no. OAAN01132), anti-MT-CO2 (ABclonal, no. A11154), anti-COX6B1 (ABclonal, no. A2228), anti-ATP5F1C (ABclonal, no. A15257), anti-ATP6VID (ABclonal, no. A12940), anti-ATP5F1A (ABclonal, no. A11217), anti-ACLY (ABclonal, no. A3719), anti-IDH3A (ABclonal, no. A14650), anti-DLD (ABclonal, no. A13296), anti-ACO2 (ABclonal, no. A3716), anti-MDH2 (ABclonal, no. A13516), anti-ENO2 (Cell Signaling, no. 9536), anti-TPI (ABclonal, no. A15733), anti-MPC1 (Cell Signaling, no.14462), and anti-MPC2 (Cell Signaling, no. 46141). After washing, membranes were incubated with secondary antibodies at a 1:5000 dilution for 2 h at room temperature. After washing, membranes were developed using the Cytiva Amersham ECL Prime Western Blotting Detection Reagent and scanned. Ponceau S stained membrane was used as total protein loading control. When necessary, membranes were stripped using Restore PLUS Western Blot Stripping Buffer (Thermo Fisher Scientific #46430) according to the manufacturer’s instructions to remove bound antibodies, and then reblocked and reprobed with a different primary antibody. Immunoblot bands from five biological replicates in both control and irradiated groups were analyzed and quantified using ImageJ software (http://rsbweb.nih.gov/ij/).

### Statistical Analysis

All experiments were conducted in five biological replicates. Data were presented as mean with standard deviation, and statistical analyses were conducted using GraphPad Prism 9.0 software. Normal distribution was evaluated using the D’Agostino-Pearson omnibus normality test. Statistical significance for comparisons between two groups was determined using the nonparametric Mann–Whitney test. Differences were considered significant if p-values were <0.05.

## Results

### Proteome Profiling of Cerebral Microvessel after RT

To investigate the effects of radiation on cerebral microvasculature, we performed quantitative proteomics using LC-MS/MS on samples from male mice six months after RT with 12 Gy X-ray or sham treatment **(** Figure 1 **A)**. The full workflow for this proteomics study is shown in Figure 1B. A total of 7,462 proteins were detected, and 2,457 were quantified **(see** Table S1, S2 **)**. Label-free quantitation (LFQ) demonstrated reproducibility among biological replicates from both control and irradiated mice, as shown by Pearson correlation **(** Figure 1 **C)**. Principal component analysis (PCA) using LFQ intensity values of all identified proteins revealed a clear separation between control and irradiated groups, indicating significant changes in protein expression due to radiation **(** Figure 1 **D)**.

**Figure 1:**
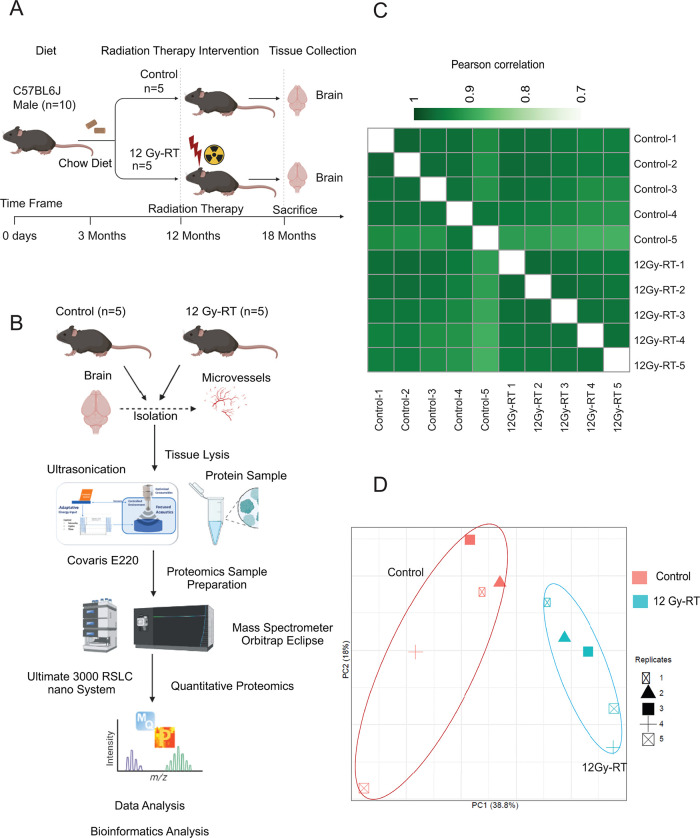
Quantitative proteomics analysis of the cerebral microvessels. (A) Workflow for radiation therapy (RT) and sample collection. (B) Workflow for high-throughput identification and quantification of proteins from cerebral microvessels in control and irradiated mice (12 Gy-RT). (C) Pearson correlation matrix based on label-free quantification (LFQ) intensities, showing sample reproducibility in control and irradiated groups. (D) Principal component analysis (PCA) plot for proteins identified in biological replicates of control and irradiated groups.

Proteins were classified as identified with at least one valid MS/MS spectrum, quantified if detected in all five biological replicates, and dysregulated if their relative abundance significantly differed between the groups. Among the 2,457 quantified proteins, 414 proteins were significantly dysregulated between irradiated and control groups, and these proteins were used for further bioinformatics analysis. Hierarchical clustering of z-scored LFQ intensities of dysregulated proteins highlighted substantial expression differences between the groups **(** Figure 2 **A)**. Of the 414 dysregulated proteins, 157 were upregulated, and 257 were downregulated (see Table S3). Given the small sample size, we calculated Cohen’s d for all statistically significant proteins to better understand the strength of the observed changes. Our analysis showed that many of the proteins had large effect sizes (Cohen’s *d* > 1.0), supporting that the differences were statistically significant and also biologically meaningful. In several cases, proteins with moderate fold changes still showed large Cohen’s d values, indicating strong and consistent differences between groups even with a modest sample size. Volcano plots depicting these changes, with the top 10 proteins in each category labeled, are shown in Figure 2B. Our microvessel isolation likely includes multiple cell types beyond endothelial cells, including pericytes and astrocytic end feet. To assess the cellular composition, we compared our full proteomics data set (2,457 quantified proteins) with a reference single-cell transcriptomic atlas of the mouse brain vasculature^40^ (Single Cell RNA-seq Gene Expression Data). This analysis revealed that 127 marker proteins were specifically enriched for endothelial cells, 97 marker proteins for pericytes, and 63 marker proteins for astrocytes, confirming a heterogeneous cellular profile (see Table S4). Among these, 7 endothelial specific proteins (Cd34, Dpp4, Lpcat4, Slc6a6, Lyn, Col18a1, Ttr), 12 pericyte specific proteins (Itga4, Rapgef5, Gjc1,Tfpi, Arhgdib, Egflam, Gja4, Atp2a3, Cpm, Myo1b, Mgp,Tgfb1i1) and 23 astrocyte specific proteins (Slc6a1, Gabbr2, Bcan, Aldh1l1, Ncam1, Btbd17, Cntn1, Dclk1, Myh14, Gpm6a, Bdh1, Soga3, Ogdhl, Sfxn5, Plp1, Phgdh, Hapln1, Tst, Ckb, Slc27a1, Mapt, Cpe, Rtn1) were dysregulated at 6 months postradiation exposure. Gene ontology (Biological Process) analysis of endothelial cell-specific dysregulated proteins revealed enrichment in pathways related to regulation of cell adhesion mediated by integrins (GO:0033638), hemopoiesis (GO:0030097), and positive regulation of cellular processes (GO:0048522). Dysregulated pericyte-specific proteins were associated with negative regulation of protein binding (GO:0032091), while dysregulated astrocyte-specific proteins were enriched in processes such as central nervous system development (GO:0007417), synapse organization (GO:0050808), and brain development (GO:0007420).

**Figure 2:**
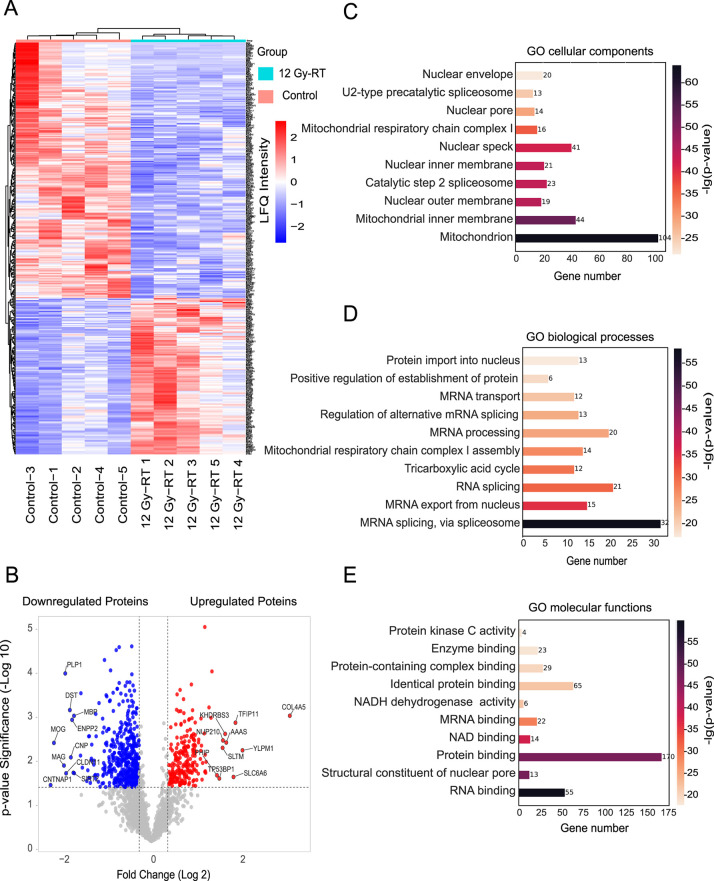
Significant differences in the cerebral microvessel proteomes between control and irradiated groups. (A) Hierarchical clustering analysis (heatmap) using unsupervised Euclidean distance for differentially expressed proteins across biological replicates. Z-Scored LFQ intensities for individual mice are shown side by side. (B) Volcano plots of differentially expressed proteins, with the *y*-axis representing a −log10 *p*-value significance and the *x*-axis representing log2 fold change. The top 10 proteins with the most significant decrease and increase in expression are highlighted in blue and red, respectively (*p* < 0.05). The dotted line indicates the cutoff for protein expression fold change (log2) against statistical significance [−log10 (*p*-value)]. (C) GO cellular components, (D) GO biological processes, and (E) GO molecular function analysis of significantly dysregulated proteins compared to the control group. The top 10 enriched GO terms are displayed with corrected p-values and gene numbers. The colored bar indicates enriched terms with corresponding corrected *p*-values and gene numbers. Analyses were performed using the HemI 2.0 web tool.

To investigate the biological significance of the dysregulated proteins, we utilized the Helm webtool for further analysis. The highest representation of dysregulated proteins was observed in the cellular components “mitochondrion”, “mitochondrial inner membrane”, and “nuclear speck”, a specialized subnuclear structure associated with splicing factors (Figure 2C). Regarding biological processes, proteins involved in mRNA splicing and processing, as well as the TCA cycle and mitochondrial respiratory chain complex I assembly, were predominantly affected (Figure 2D). For molecular function, dysregulated proteins were primarily associated with protein and RNA binding activities (Figure 2E). A complete list of these categories and their corrected p-values is provided in Table S5.

### Impact Pathway Analysis

In addition, we analyzed all changes with iPathwayGuide that unlike other functional analysis tools, considers a gene’s position within each pathway and its interactions with other genes. The analysis revealed that the significantly dysregulated proteins were enriched in 266 pathways, with strongest impact on metabolic pathways, nucleocytoplasmic transport, one carbon metabolism, oxidative phosphorylation, spliceosome, and the tricarboxylic acid cycle **(** Figure 3 **A-F).**

**Figure 3:**
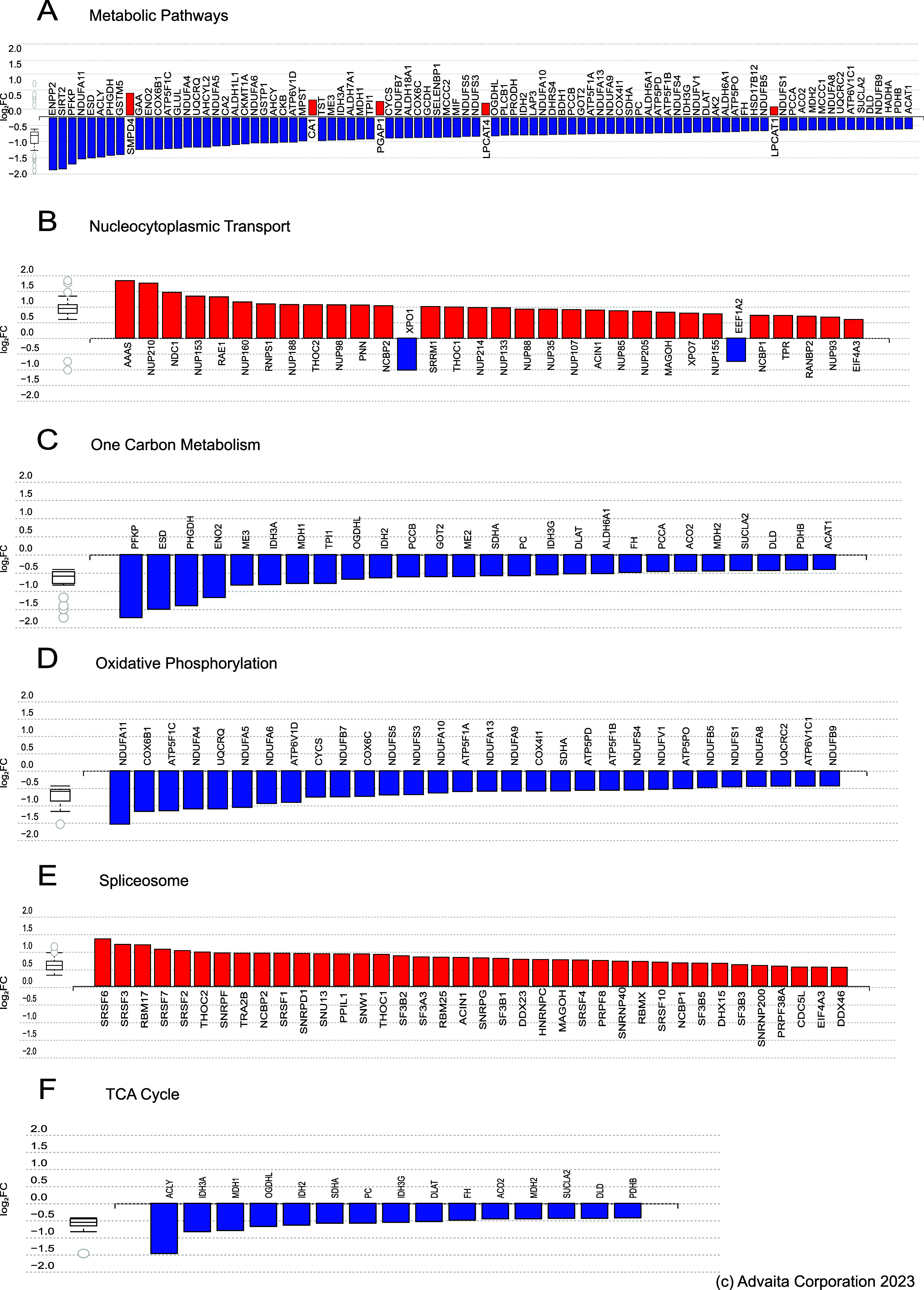
Impact pathway (iPathway guide) analysis of dysregulated proteins by RT. (A–F) Bar graphs showing dysregulated proteins mapped to pathways identified by iPathway analysis based on FDR-corrected *p*-value significance: (A) metabolic pathways, (B) nucleocytoplasmic transport, (C) one carbon metabolism, (D) oxidative phosphorylation, (E) spliceosome, and (F) TCA cycle. Genes are ranked by absolute log-fold change, with upregulated genes in red and downregulated genes in blue. Box-and-whisker plots summarize the distribution of differentially expressed genes in each pathway, with boxes representing the first quartile, median, and third quartile, and circles indicating outliers.

Additionally, several nonmetabolic pathways were significantly enriched in irradiated cerebral microvessels, including cGMP-PKG signaling, vascular smooth muscle contraction, mRNA surveillance, calcium signaling **(** Figure S1 A-E **)**, focal adhesion, tight junction, and gap junction, **(** Figure S2 A-C **)**. Moreover, proteins enriched in the spliceosome and mRNA surveillance pathways were upregulated in irradiated group, potentially suggesting an increased stress response and errors in mRNA processing due to radiation exposure. A complete list of enriched pathways and their corrected p-values is available in Table S6.

The core analysis module in the Ingenuity Pathway Analysis (IPA) software was used to identify altered canonical pathways in cerebral microvessels following RT. Using a -log (p-value) cutoff of 1.3 (Fisher’s exact test) and a z-score threshold (Z-score >2 for activation, Z-score < −2 for inhibition), 76 enriched canonical pathways were identified. Of these, 68 pathways were predicted to be inhibited and 8 activated post-RT. Figure 4 **A-C** and Table S7 highlight 15 canonical pathways associated with cerebral microvessel function. Based on altered protein expression, the three most significantly inhibited pathways were oxidative phosphorylation, the TCA cycle, and Gαq signaling, while the five most significantly activated pathways included mitochondrial dysfunction, sirtuin signaling, granzyme A signaling, the spliceosomal cycle, and RHOGDI signaling.

**Figure 4:**
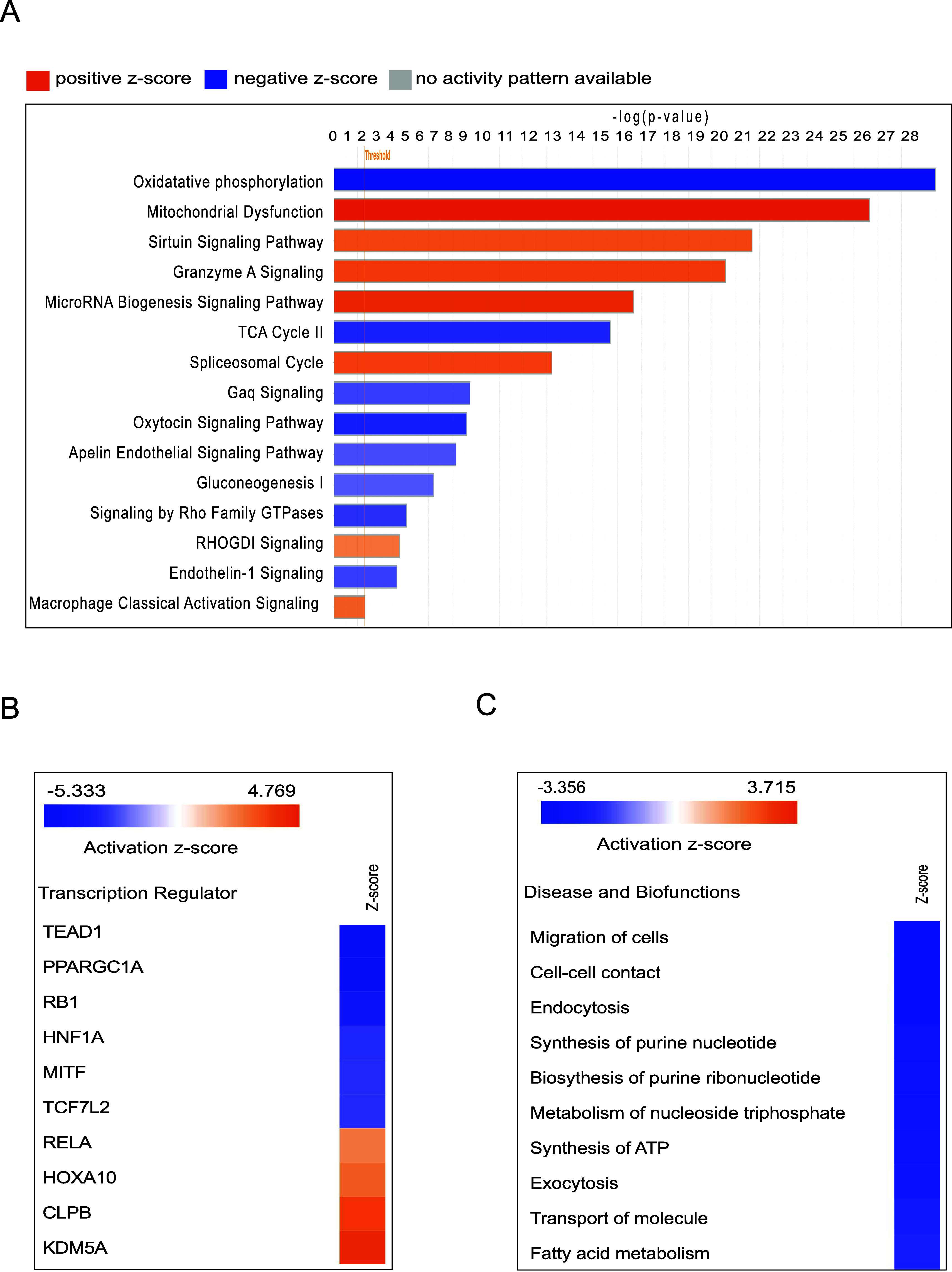
Ingenuity pathway analysis (IPA) of all dysregulated proteins from the irradiated group. (A) Fifteen selected canonical pathways predicted to be activated or inhibited based on z-scores and a B–H *p*-value of <0.05 (calculated using Fisher’s exact test and adjusted with the Benjamini–Hochberg method). A positive z-score (orange) indicates pathway activation, while a negative z-score (blue) indicates inhibition. Longer bars indicate stronger significance (source: http://www.INGENUITY.com). (B) Heatmap of the top 10 transcription regulators predicted to be activated (z-scores > 2) or inhibited (z-scores < −2). (C) Predicted activation or inhibition of selected diseases and biofunctions based on z-scores (>2 for activation; <−2 for inhibition) in the irradiated group.

An upstream regulator analysis using the z-score algorithm identified 34 enriched transcription regulators in irradiated cerebral microvessels. The top activated regulators were KDM5A (Z-score = 4.243), CLPB (Z-score = 3.742), HOXA10 (Z-score = 2.714), and RELA (Z-score = 2.201), while the top inhibited regulators included TEAD1 (Z-score = −4.899), PPARGC1A (Z-score = −4.872), RB1 (Z-score = −3.90), HNF1A (Z-score = −2.985), and MITF (Z-score = −2.887) (Figure 4B). A disease and biofunction analysis of 414 differentially expressed proteins revealed enrichment for 41 diseases and biological functions postradiation exposure. Of these, 31 functions were predicted to be inhibited, while 10 were activated. Figure 4C depicts the selected 10 enriched diseases/biological functions, associated molecules, and their p-values and z-scores. Notable affected functions in cerebral microvessels after RT included cell migration, cell-to-cell contact, ATP synthesis, molecule transport, and fatty acid metabolism, as detailed in Table S7.

### RT Induces Changes in the Expression of Mitochondrial Proteins within Cerebral Microvessels

Given the strong evidence for changes in the mitochondrial proteome by different analysis tools, we investigated the impact of RT on mitochondrial protein expression and associated functions in more detail. We mapped all 2,467 quantified cerebral microvessel proteins to the Mouse Mitocarta3.0 database. This analysis identified 385 mitochondrial proteins, with 84 significantly dysregulated (all downregulated) in the irradiated group compared to controls **(** Figure 5 **A;** Table S8 **)**. Volcano plots for mitochondrial proteins depicting these changes, with the top 10 downregulated proteins are labeled, are shown in Figure 5 **B.** We created a hierarchical cluster heatmap using the LFQ intensity of significantly dysregulated mitochondrial proteins, clearly separating control and irradiated groups (Figure 5C).

**Figure 5:**
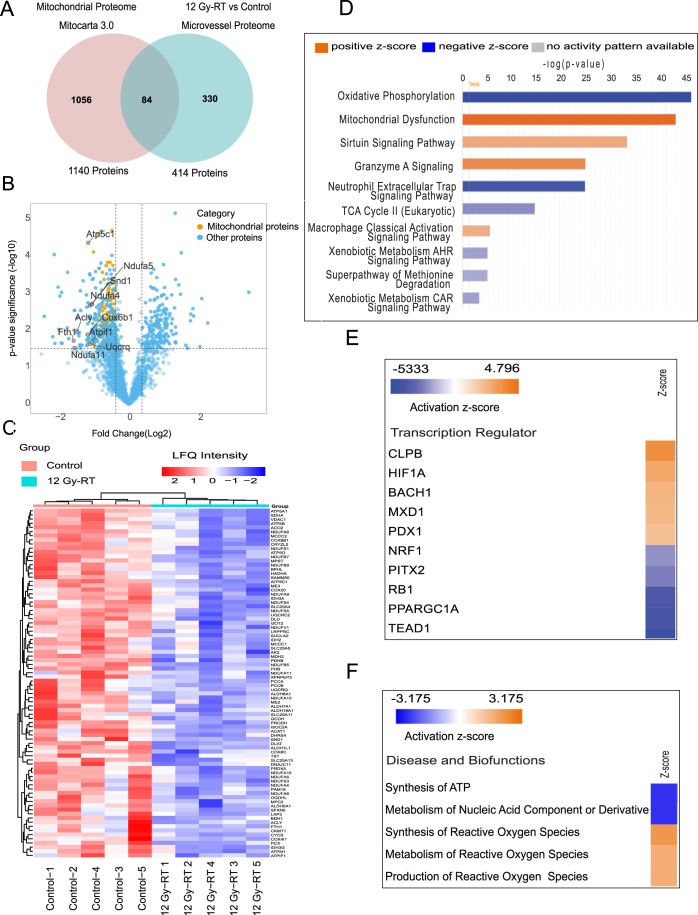
Ingenuity pathway analysis (IPA) of mitochondrial proteins that are dysregulated in the irradiated group. (A) Venn diagram showing 84 mitochondrial proteins shared between the mitochondrial proteome database (Mitocarta 3.0) and the cerebral microvessel proteome. (B) Volcano plot of differentially expressed mitochondrial proteins in irradiated group (top 10 proteins are labeled). (C) Top 10 predicted canonical pathways based on z-scores and B–H *p*-values <0.05 (calculated using Fisher’s exact test and adjusted with the Benjamini–Hochberg method). A positive z-score (orange) indicates pathway activation, while a negative z-score (blue) indicates inhibition (source: http://www.INGENUITY.com). (D) Heatmap of the top 10 transcription regulators predicted to be activated (z-scores > 2) or inhibited (z-scores < −2). (E) Predicted activation or inhibition of selected diseases and biofunctions based on z-scores (>2 for activation; <−2 for inhibition) in the irradiated group.

To confirm the biological significance of these changes and their impact on mitochondrial function, we performed gene ontology (GO) analysis with the Helm webtool. The most affected proteins were linked to the mitochondrial inner membrane, mitochondrial respiratory chain complex I, and mitochondrial matrix (Figure S3 A, Table S9). Key impacted biological processes included mitochondrial respiration, the tricarboxylic acid cycle, and 2-oxoglutarate metabolic process (Figure S3 B).

Using IPA core analysis, we examined affected canonical pathways in the mitochondrial protein data set. This analysis identified 14 enriched pathways, 10 predicted to be inhibited and 4 activated following radiation treatment. The top three inhibited pathways were oxidative phosphorylation, neutrophil extracellular trap signaling, and the TCA cycle (Figure 5D). Additionally, upstream regulator analysis for transcription factors associated with mitochondrial proteins identified 14 enriched regulators. Among those, CLPB, and HIF1A were the top activated regulators, while TEAD1, PPARGC1A, and RB1 were the most inhibited (Figure 5E). Table S10 provides a comprehensive list of canonical pathways, upstream regulators, and associated p-values. Lastly, we used IPA software to map altered diseases and biological functions based on dysregulated mitochondrial proteins. The analysis highlighted 22 enriched diseases and functions, 17 predicted to be inhibited and 5 activated. Notably, ATP synthesis and nucleic acid metabolism were predicted to decrease, while synthesis, production and metabolism of reactive oxygen species were predicted to increase (Figure 5F).

### Metabolic Pathways Are Affected in Cerebral Microvessels after RT

Energy metabolism is essential for biosynthetic processes to facilitate the restoration of cell damage. While previous studies have extensively explored the impact of radiation on cardiac energy metabolism^35,44,45^ and metabolic pathways in other tissues^46,47^ the impact of radiation on cerebral microvessel metabolism is less understood. Our proteomic analysis revealed altered expression of proteins linked to key metabolic pathways, including oxidative phosphorylation (n = 29), the TCA cycle (n = 15), and glycolysis (n = 7) (Figure S4 A-C). IPA analysis suggested inhibition of oxidative phosphorylation, the TCA cycle, glycolysis, and gluconeogenesis. To confirm these findings, we used Western blotting to analyze dysregulated proteins in oxidative phosphorylation, the TCA cycle, and glycolysis.

Our analysis revealed radiation-induced changes in the expression of proteins across various OXPHOS system complexes. Western blot images for different OXPHOS protein subunits with adjustment for loading by Ponceau-stained whole membranes **(** Figure S5 A-E **)** revealed significant downregulation of complex I proteins NDUFA11 and NDUFB8 (Figure 6 **A-C**), complex II protein SDHB (Figure 6 **A-C**), and complex IV protein MT-CO2 (Figure 6 **A-C**) in irradiated samples. Although downregulation of complex III and V proteins ATP5F1C, ATP6VID, and ATP5F1A was observed, these changes did not reach statistical significance (Figure 6 **A-C**, Figure S6 A–D **).**

**Figure 6:**
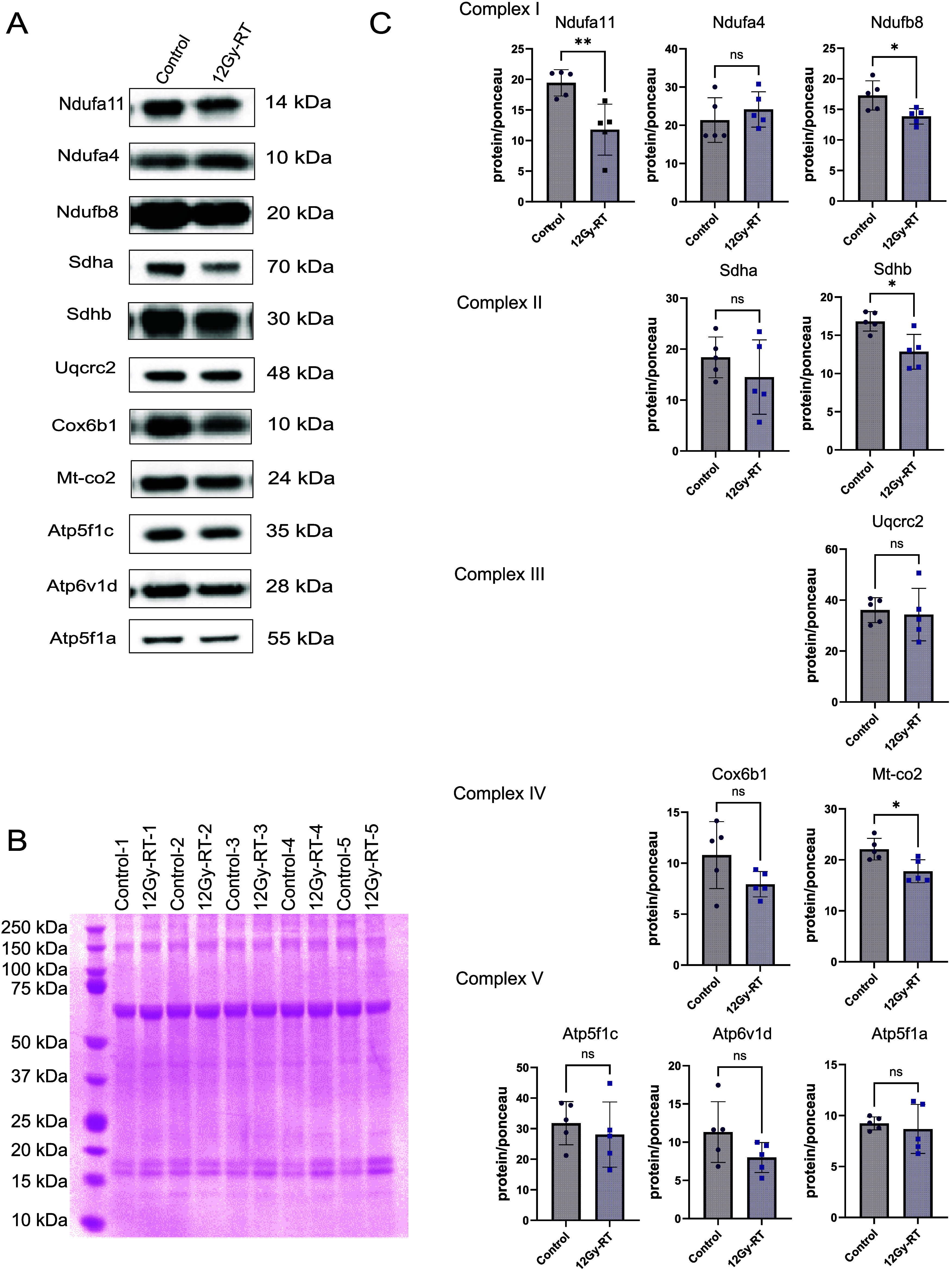
Validation of metabolic protein expression changes by immunoblot. (A) Representative immunoblot images for OXPHOS complex subunits in control and irradiated groups. (B) Ponceau-stained membrane used as loading control. (C) Immunoblots of ETC complex I proteins (NDUFA11, NDUFA4, and NDUFB8), complex II proteins (SDHA and SDHB), complex III protein (UQCRC2), complex IV proteins (COX6B1 and MT-CO2), and complex V proteins (ATP5F1C, ATP6VID, and ATP5F1A) in control and irradiated groups. Quantification of protein levels after normalization to Ponceau-stained membrane. Analysis by the Mann–Whitney test.

Lastly, we examined proteins involved in the TCA cycle, glycolysis, and pyruvate transport proteins and detected nonsignificant trends toward decreases for TCA cycle proteins UQCRC2, ACLY, ACO2, IDH3A, DLD, SDHA, and MDH2 in the irradiated group (Figure S 7A-B). Similarly, analysis of glycolysis and pyruvate transport proteins revealed similar trends for glycolytic proteins TPI and ENOL2 as well as mitochondrial pyruvate carrier proteins MPC1 and MPC2 in the irradiated group (Figure S 7C–D and Figure S 8A-B).

## Discussion

Our study provides the first in-depth analysis of long-term proteome changes in nontumor cerebral microvessels following RT in C57Bl6J mice, offering key insights into the impact of radiation on vascular health. A total of 414 dysregulated proteins were identified, with 257 showing downregulation. These findings highlight mitochondrial dysfunction and metabolic disruptions as central themes, with notable effects on pathways such as oxidative phosphorylation, the TCA cycle, and glycolysis.

Pathway enrichment analysis revealed significant alterations in 266 pathways. Among 76 canonical pathways, 68 were inhibited (e.g., oxidative phosphorylation), while 8 were activated (e.g., mitochondrial dysfunction). Mitochondrial proteins were particularly impacted, with a detailed analysis identifying 84 downregulated proteins linked to the mitochondrial inner membrane and respiratory chain complex I, indicating disrupted energy metabolism.

Proteomic changes also revealed inhibition of ATP synthesis, cell migration, and other cellular processes through pathways such as nucleo-cytosolic transport and spliceosomal activity. Increased oxidative stress was evidenced by the activation of reactive oxygen species synthesis, potentially as a result of mitochondrial dysfunction. Enriched disease-related processes further pointed to disruptions in molecule transport and fatty acid metabolism. Western blot analysis of OXPHOS proteins confirmed inhibited energy metabolism and mitochondrial dysfunction. These findings underscore the lasting effects of RT caused by RT in cerebral microvessels, particularly on mitochondrial health and metabolic pathways.

Given the strong impact of RT on the mitochondrial proteome, our study focused on changes in the mitochondrial and metabolic proteome. In most vascular beds, endothelial cells (ECs) predominantly use glycolysis and depend minimally on mitochondrial OXPHOS for ATP generation, as mitochondrial volume in ECs constitutes only 2–6% of total cellular volume.^48,49^ However, cerebromicrovascular ECs at the BBB possess nearly double the mitochondrial volume compared to other vascular beds.^50^ The BBB represents a selective yet dynamic interface between the blood and central nervous system that rigorously maintains neuronal homeostasis by regulating the transport of substances to and from the brain.^51,52^ The cerebral microvasculature engages in aerobic respiration to support the energy demands required for maintaining transport systems and barrier function.^53^ Doll and colleagues reported that mitochondrial “crisis” induced by a lipopolysaccharide challenge in cerebromicrovascular ECs significantly compromises BBB function in vitro and in vivo^52^ but similar data after RT have been missing so far.

Since glycolysis is the primary metabolic pathway for energy production in ECs, it remains to be established how the extensive alterations in metabolic function following RT affect EC phenotypes. As such, further studies are needed to explore the positive link between energy depletion and EC dysfunction, for example, whether energy deficiency drives or results from long-term changes post-RT, including altered BBB permeability, cellular senescence, impaired angiogenesis, increased inflammation, reduced nitric oxide production, and a pro-thrombotic state.^48,54–58^ Since these changes may contribute to complications like neuroinflammation and ischemia in irradiated areas, largely due to the disruption of vascular homeostasis, targeting metabolic pathways may offer a novel approach to mitigating these long-term side effects.

Beyond metabolism, mitochondrial signaling is gaining recognition for its role in RT-induced tissue injury.^59–62^ While mitochondrial reactive oxygen species (ROS) have been highlighted as mediators of mitochondrial DNA damage and ETC dysfunction in ECs, additional factors, such as altered mitochondrial calcium in- and efflux, may also contribute.^26,61,63,64^ Moreover, mitochondrial DNA fragments as damage-associated molecular patterns may drive inflammatory signaling cascades after RT.^65^

Additional to changes in the mitochondrial and metabolic proteome, our findings highlight dysregulation in proteins related to mRNA splicing and stress-response pathways, suggesting that RT drives transcriptional and post-transcriptional modifications.^66^ These changes potentially disrupt cellular homeostasis and protein synthesis, aggravating vascular dysfunction and warrant further investigation.

This study has several limitations. First, exclusively male mice were used. This was done to reduce variability due to the during the estrous cycle. However, given emerging evidence for differences in radiosensitivity in males versus females, findings should be validated in female mice as well,^67^ to understand sex-dependent differences in radiation response particularly in microvascular injury and cognitive outcomes. Second, the current bulk proteomics approach does not allow for the resolution of contributions from distinct vascular-associated cell types, such as pericytes, astrocytes, and endothelial cells. Advanced techniques, including single-cell proteomics, spatial transcriptomics, or fluorescence-activated cell sorting (FACS) of purified cell populations, will be essential for achieving higher-resolution, cell-type-specific insights into radiation-induced vascular injury responses. Third, our observations were limited to a single postradiation time point and a modest sample size. Future studies with multiple time points and larger cohorts are needed to capture the temporal dynamics of microvascular injury and to improve sensitivity for detecting subtle changes in protein expression following radiation exposure. Lastly, mitochondrial cell lysate preparation, additional validation and mechanistic follow-up studies are necessary to confirm the proteomics data and bioinformatics predictions, particularly those related to mitochondrial function, blood-brain barrier integrity, and RNA processing pathways.

In conclusion, this study provides the first evidence of radiation-induced proteomic alterations in the cerebral microvasculature, including mitochondrial dysfunction, disruption of metabolic pathways and dysregulation of vascular signaling pathways. To our knowledge, this is the first investigation to employ a quantitative proteomics approach to characterize microvascular changes in the brain following RT. These findings shed light on potential mechanisms underlying radiation-induced BBB breakdown and highlight the need for further investigation into the role of mitochondria in endothelial cells and other vascular-associated cell types. They also underscore the need for further research to understand the interplay between mitochondrial integrity, cellular metabolism and vascular health in the context of radiation exposure.

## Supplementary Material





## Data Availability

The mass spectrometry proteomics data have been deposited in the ProteomeXchange Consortium via the PRIDE repository with the data set identifier PXD058732. All data are available in the main text or the Supporting Information.

## Notes

(Table S1) Proteomics results displaying the list of all Identified proteins from control and irradiated cerebral microvessels; (Table S2) list of all quantified proteins from control and irradiated cerebral microvessels and proteins with two unique peptides in bold; (Table S3) list of all significantly dysregulated proteins in the irradiated group with two unique peptides and showing a fold-change ratio of <0.77 or >1.30, with corrected *p*-values (*q*-value significant+) and Cohens’ *d* value; (Table S4) analysis of cell type specific proteins in the microvasculature proteome; (Table S5) GO ontology analysis of dysregulated proteins from the irradiated cerebral microvessels; (Table S6) list of all impacted pathways identified using iPathwayGuide tool; (Table S7) list of activated (orange color) and inhibited (blue color) canonical pathways, upstream regulators, disease, and biofunctions with overlapped molecules in the irradiated cerebral microvessels; (Table S8) list of all significantly dysregulated mitochondrial proteins in the irradiated group; (Table S9) GO ontology analysis of dysregulated mitochondrial proteins from the irradiated cerebral microvessels; (Table S10) list of activated (orange color) and inhibited (blue color) canonical pathways, upstream regulators, disease, and a selected list of biofunctions with overlapped molecules in the mitochondria of irradiated cerebral microvessels (XLSX)
(Figure S1) Impact pathway (iPathway guide) analysis of dysregulated proteins in various pathways; (Figure S2) impact pathway (iPathway guide) analysis of dysregulated proteins in cell–cell contact pathways; (Figure S3) Gene Ontology (GO) enrichment analysis of dysregulated mitochondrial proteins; (Figure S4) impact pathway (iPathway guide) analysis of dysregulated proteins involved in OXPHOS, TCA cycle, and glycolysis; (Figure S5) Ponceau-stained whole membrane as loading control for Western blotting; (Figure S6) uncropped immunoblots of electron transport chain proteins; (Figure S7) immunoblot validation of TCA cycle, glycolysis, and pyruvate transport proteins; (Figure S8) uncropped immunoblots of TCA cycle, glycolysis, and pyruvate transport proteins (PDF)

## †

Department of Internal Medicine, University of Oklahoma College of Medicine, 6444 AAT, 800 Stanton L. Young Blvd., Oklahoma City, OK 73104, USA (I.M.G.)
