# Supplementary material for: Long-Term Effects of Radiation Therapy on Cerebral Microvessel Proteome: A Six Month Post-Exposure Analysis
Source: ACS Omega. 2025 Oct 26;10(43):52046–59. doi: 10.1021/acsomega.5c09726 (PMC12593089; doi:10.1021/acsomega.5c09726)
Supplement: Supplementary file 2 [file ao5c09726_si_002.pdf]

Supporting Information

Long-Term Effects of Radiation Therapy on Cerebral Microvessel Proteome: A Six-Month Post-Exposure Analysis

Vikram Subramanian [1], Denise Juhr [1], Piero Giansanti [2], Isabella M. Grumbach [1,3,4*]

[1] Abboud Cardiovascular Research Center, Department of Internal Medicine, Carver College of Medicine, University of Iowa, Iowa City 52242, Iowa, USA

[2] Bavarian Center for Biomolecular Mass Spectrometry at Klinikum rechts der Isar (BayBioMS@MRI), Technical University of Munich, Munich 81675, Germany

[3] Free Radical and Radiation Biology Program, Department of Radiation Oncology, Carver College of Medicine, University of Iowa, Iowa City 52242, Iowa, USA

[4] Iowa City VA Healthcare System, Iowa City 52246, Iowa, USA

* Current address: Isabella M. Grumbach, MD, PhD, FACC, FAHA, Professor and Chair, Department of Internal Medicine, University of Oklahoma College of Medicine, 6444 AAT, 800 Stanton L. Young Blvd., Oklahoma City, OK 73104, USA. Email: isabella-grumbach@ouhsc.edu

*** Corresponding author**:

Isabella Grumbach, MD, PhD, FACC, FAHA
Professor and Chair, Department of Internal Medicine
University of Oklahoma (OU) College of Medicine
6444 AAT
800 Stanton L. Young Blvd.
Oklahoma City, OK 73104
e-mail: isabella-grumbach@ouhsc.edu

Table of contents:

- Figure S1. Impact Pathway (iPathway guide) Analysis of Dysregulated Proteins in various pathways
- Figure S2. Impact Pathway (iPathway guide) Analysis of Dysregulated Proteins in cell-cell contact pathways
- Figure S3. Gene Ontology (GO) Enrichment Analysis of Dysregulated Mitochondrial Proteins
- Figure S4. Impact Pathway (iPathway guide) Analysis of Dysregulated Proteins Involved in OXPHOS, TCA Cycle, and Glycolysis
- Figure S5. Ponceau Stained Whole Membrane as Loading Control for Western Blotting
- Figure S6. Uncropped Immunoblots of Electron Transport Chain Proteins
- Figure S7. Immunoblot Validation of TCA cycle, Glycolysis and Pyruvate Transport Proteins
- Figure S8. Uncropped Immunoblots of TCA cycle, Glycolysis and Pyruvate Transport Proteins

Figure S1

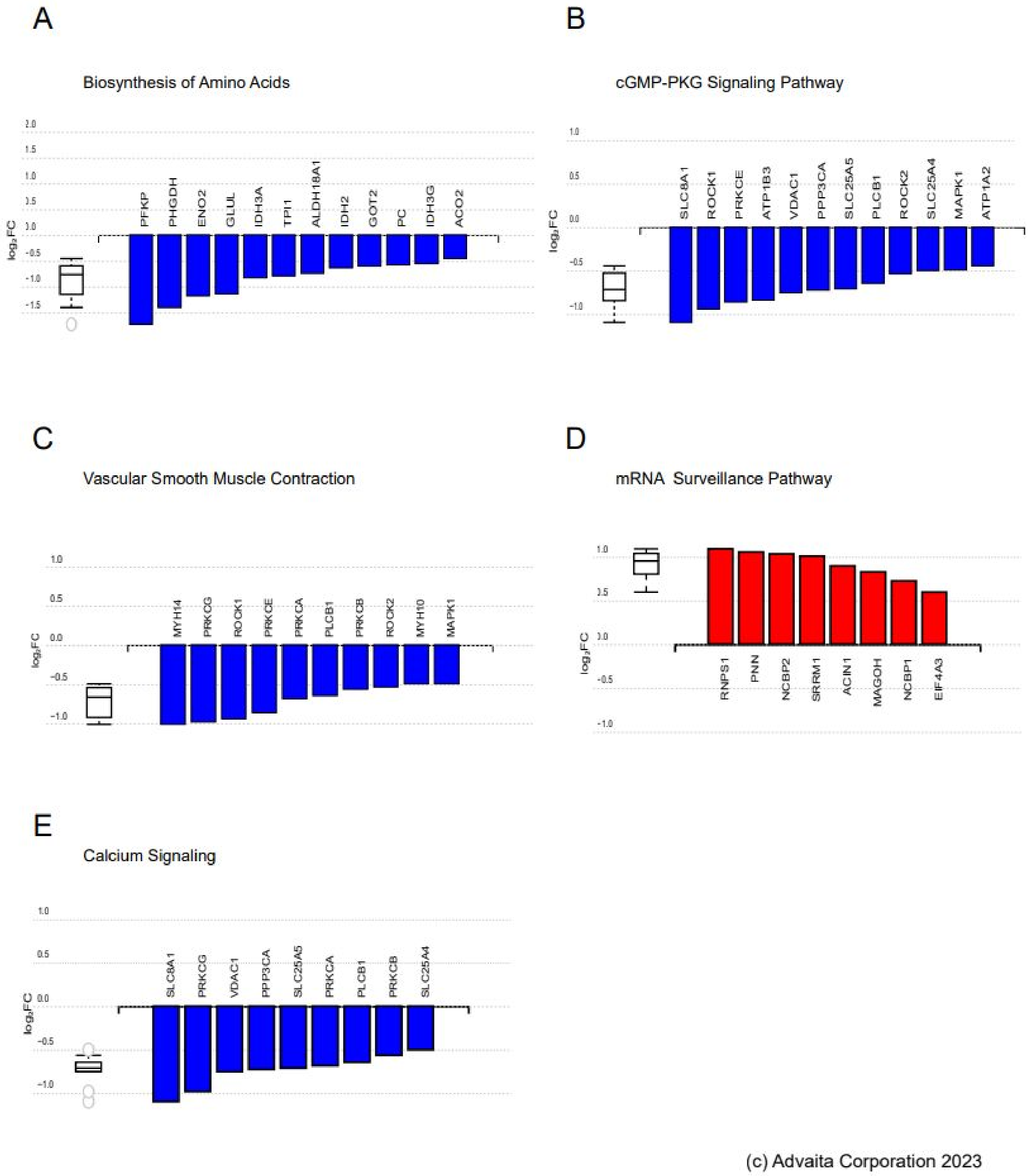

Figure S1: Impact Pathway (iPathway guide) Analysis of Dysregulated Proteins in the Irradiated Group. (A-E) Bar graphs showing dysregulated genes mapped to pathways identified by iPathway analysis based on FDR-corrected p-value significance: **(A)** Biosynthesis of amino acids, **(B)** c-GMP-PKG signaling pathway, **(C)** vascular smooth muscle contraction, **(D)** mRNA surveillance pathway, and **(E)** Calcium signaling. Genes are ranked by absolute log-fold change, with upregulated genes in red and downregulated genes in blue. Box-and-whisker plots on the

left summarize the distribution of differentially expressed genes within each pathway, with boxes representing the 1st quartile, median, and 3rd quartile, and circles indicating outliers.

Figure S2

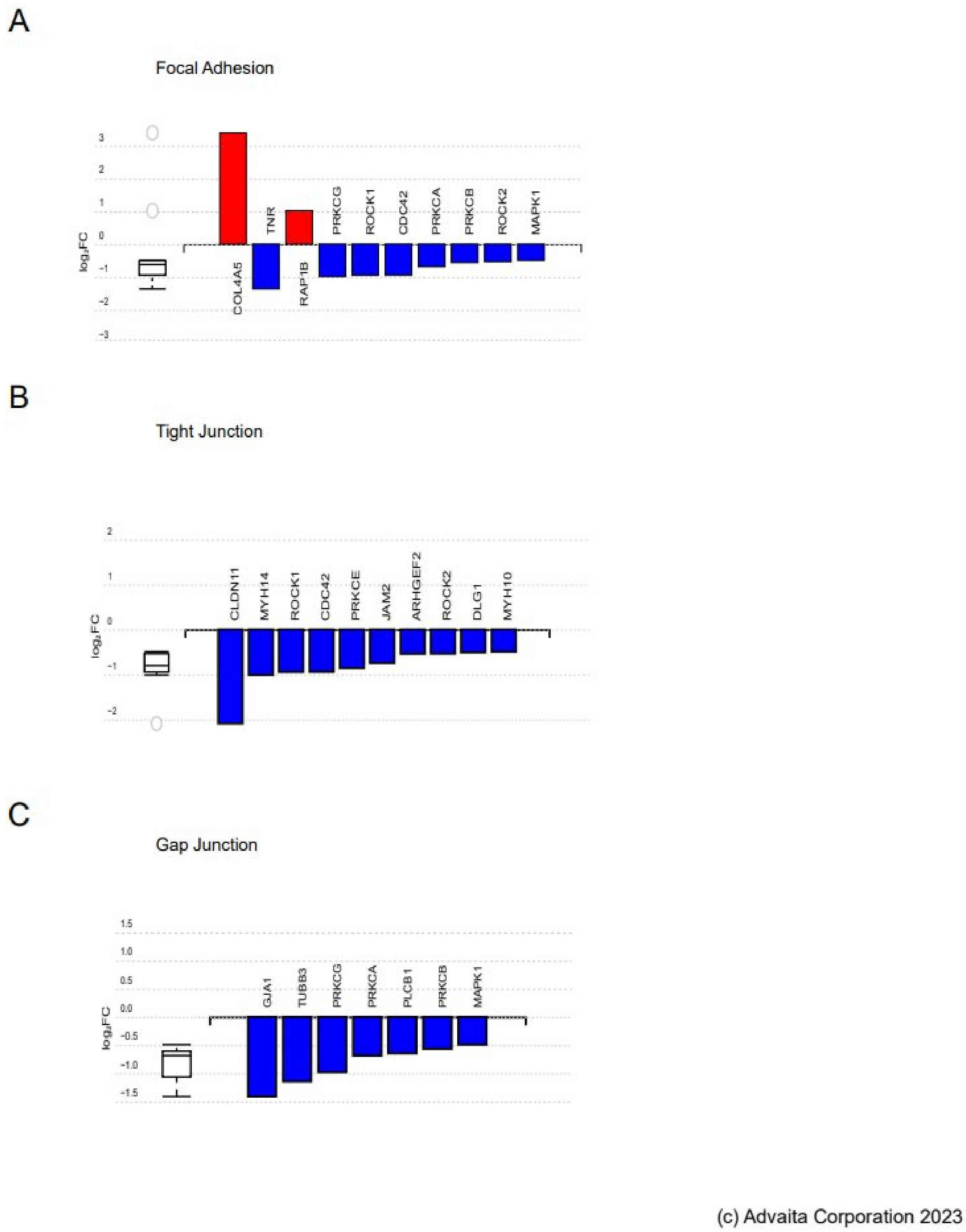

Figure S2: Impact Pathway (iPathway guide) Analysis of Dysregulated Proteins in the Irradiated Group. (A-C) Bar graphs showing dysregulated genes mapped to pathways identified by iPathway analysis based on FDR-corrected p-value significance: **(A)** focal adhesion**, (B)** tight junction**,** and **(C)** gap junction. Genes are ranked by absolute log-fold change, with upregulated genes in red and downregulated genes in blue. Box-and-whisker plots on the left summarize the distribution of differentially expressed genes within each pathway, with boxes representing the 1st quartile, median, and 3rd quartile, and circles indicating outliers.

Figure S3

A

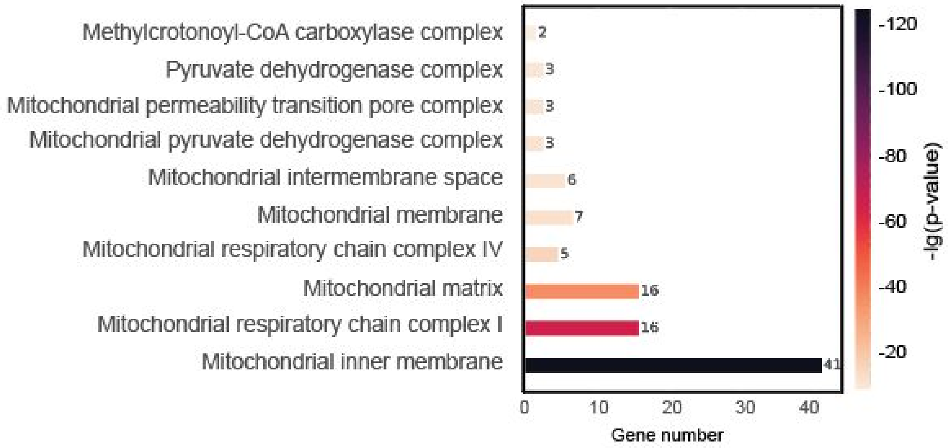

B

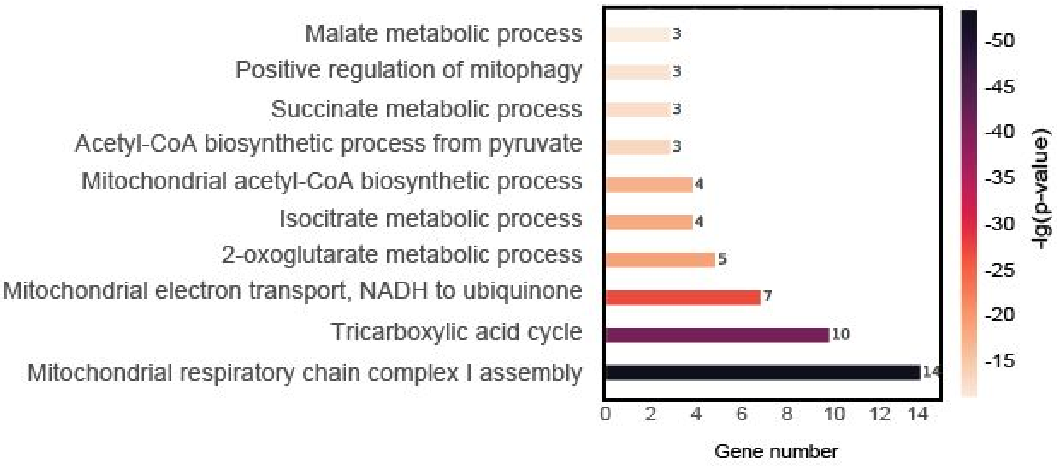

Figure S3: Gene Ontology (GO) Enrichment Analysis of Dysregulated Mitochondrial Proteins in the Irradiated Group. (A) GO cellular components and **(B)** GO biological process analyses of significantly deregulated mitochondrial proteins compared to the control group. The top 10 enriched GO terms are displayed with their corrected p-values and gene counts. The colored bar (dark to light) represents the level of enrichment, with corrected p-values and gene counts for each term. Analysis was performed using the HemI 2.0 web tool.

Figure S4

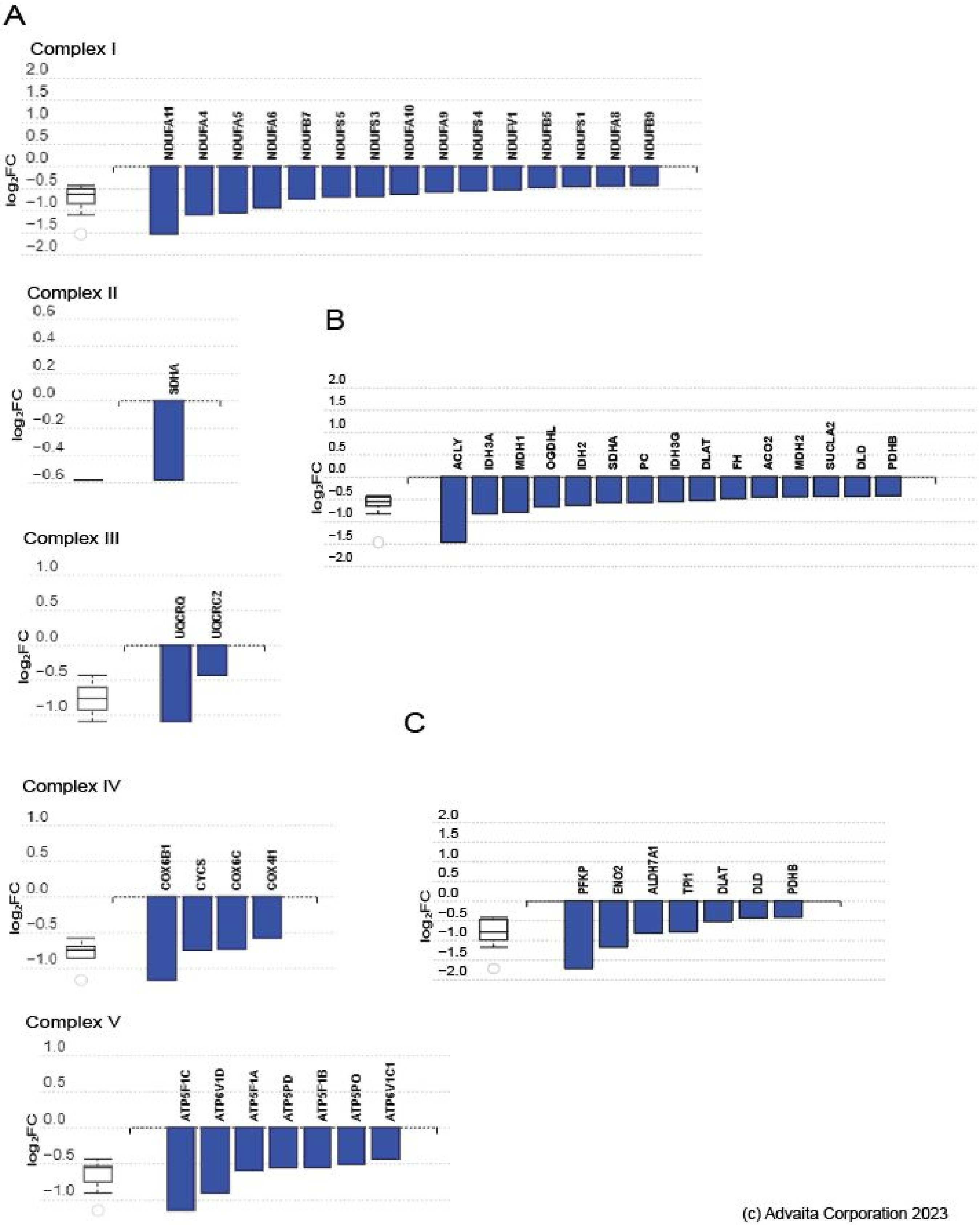

(c) Advaita Corporation 2023

Figure S4: Dysregulated Proteins Involved in OXPHOS, TCA Cycle, and Glycolysis in the Irradiated Group. (A) Bar graphs showing dysregulated genes mapped to OXPHOS, **(B)** TCA cycle, and **(C)** glycolysis identified by iPathway analysis based on FDR-corrected p-value significance and log2-fold changes. Genes or proteins in each pathway are ranked by absolute log-fold change, with upregulated genes in red and downregulated genes in blue. Box-and-whisker plots on the left summarize the distribution of differentially expressed genes within each pathway, with boxes representing the 1st quartile, median, and 3rd quartile, and circles indicating outliers.

Figure S5

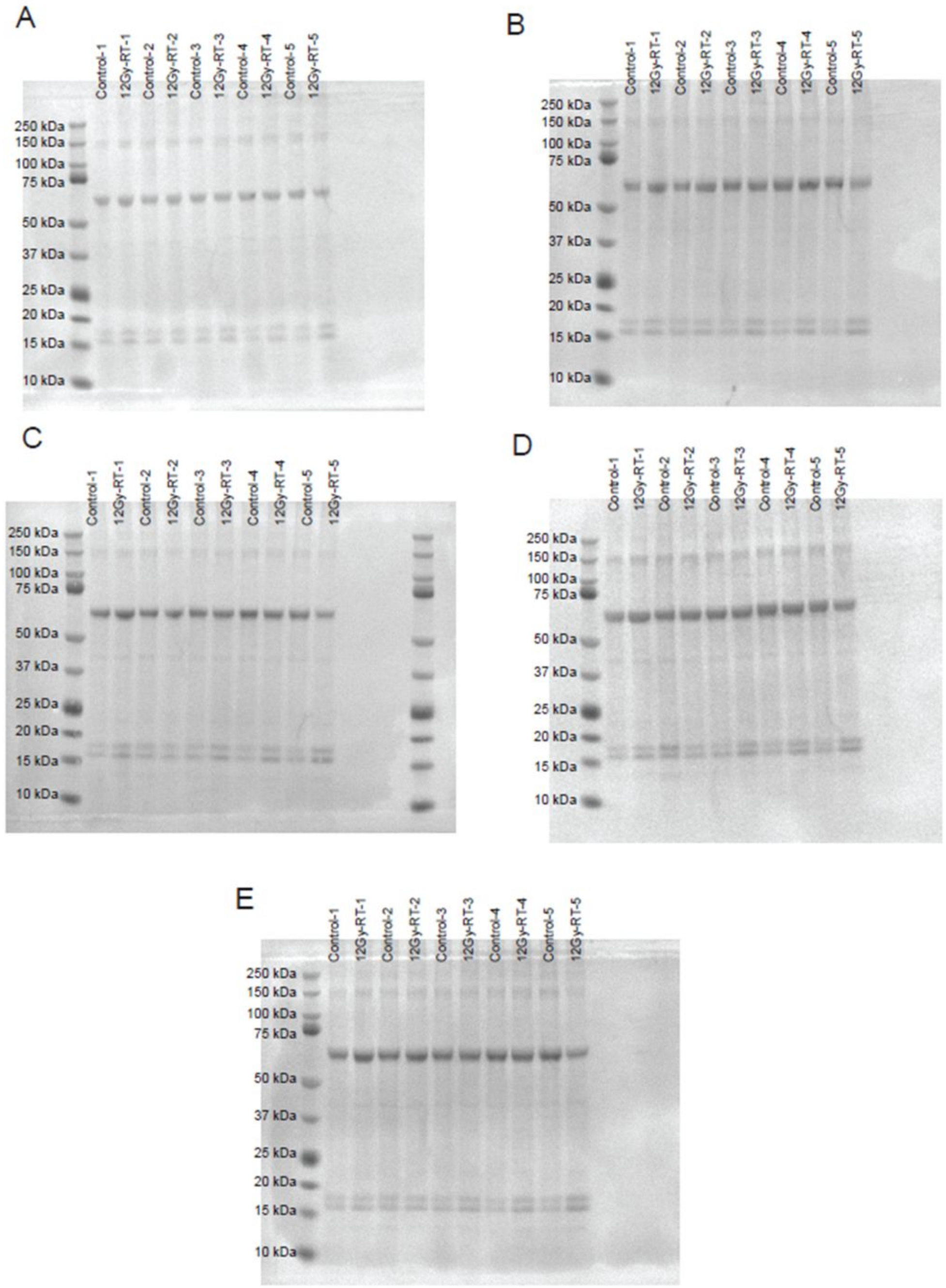

Figure S5: Ponceau Stained Whole Membrane as Loading Control for Control and Irradiated Groups. (A) Loading control for proteins (SDHA, UQCRC2, ATP5F1C). **(B)** Loading control for proteins (NDUFB8, SDHB, MT-CO2, ATP6V1D, ACO2, DLD and ENOL2). **(C)** Loading control for proteins (NDUFA4, ACLY and MDH-1). **(D)** Loading control for proteins (NDUFA11, ATP5F1A, IDH3A, TPI, MDH-2, MCP-1 and MCP-2). **(E)** Loading control for proteins (COX6B1).

Figure S6

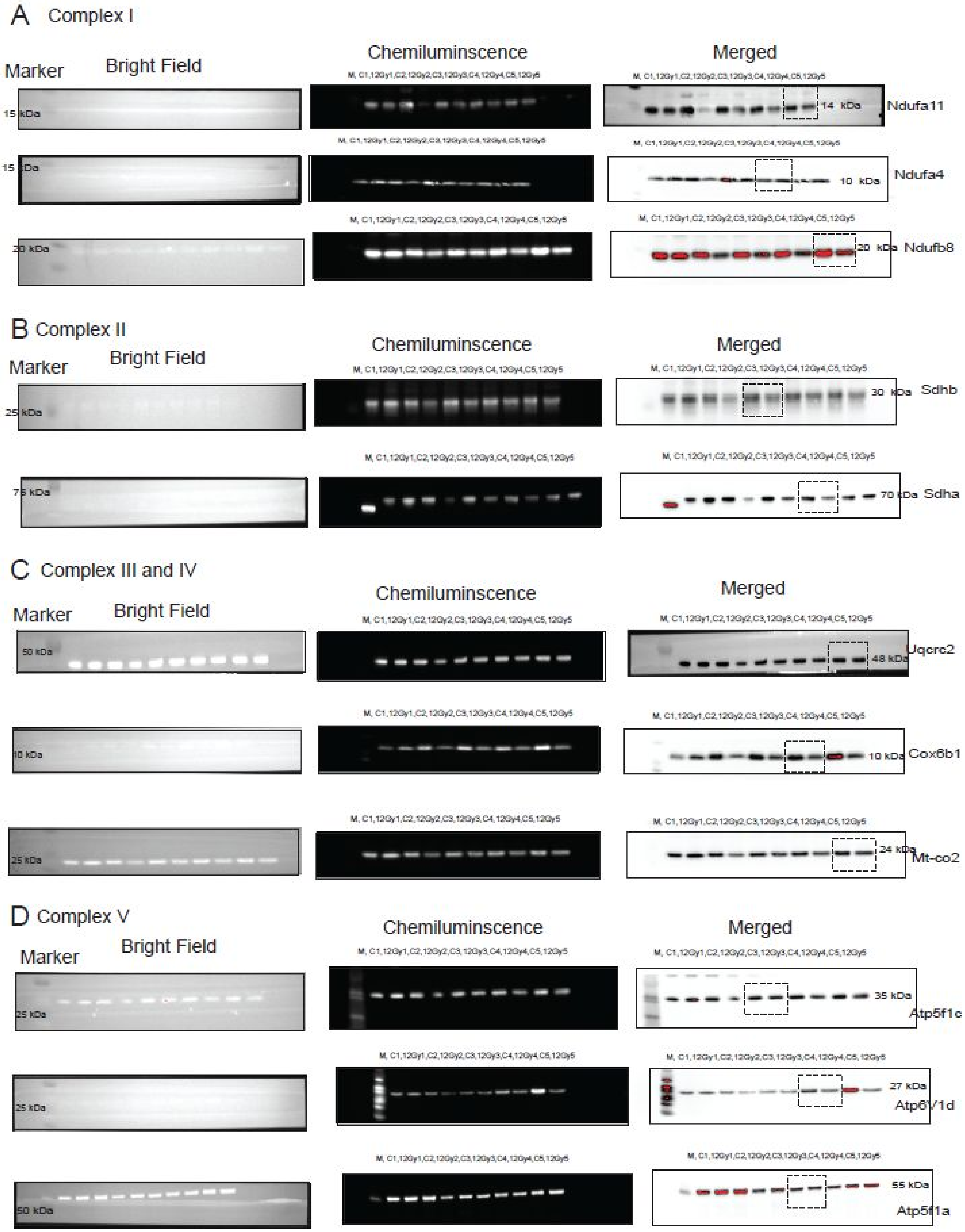
A Complex I
Marker
Bright Field
Chemiluminscence
Merged
M, C1,12Gy1,C2,12Gy2,C3,12Gy3,C4,12Gy4,C5,12Gy5
15 kDa
14 kDa
Ndufa11
15 kDa
10 kDa
Ndufa4
20 kDa
Ndufb8
B Complex II
Marker
Bright Field
Chemiluminscence
Merged
25 kDa
30 kDa
Sdhb
75 kDa
70 kDa
Sdha
C Complex III and IV
Marker
Bright Field
Chemiluminscence
Merged
50 kDa
48 kDa
Uqcrc2
10 kDa
Cox6b1
25 kDa
24 kDa
Mt-co2
D Complex V
Marker
Bright Field
Chemiluminscence
Merged
25 kDa
35 kDa
Atp5f1c
25 kDa
27 kDa
Atp6V1d
50 kDa
55 kDa
Atp5f1a

Figure S6: Uncropped Immunoblots of Figure 6. (A) Immunoblots of ETC complex I proteins (NDUFA11, NDUFA4, NDUFB8), **(B)** complex II proteins (SDHA, SDHB), **(C)** complex III protein (UQCRC2), and complex IV proteins (COX6B1, MT-CO2), **(D)** complex V proteins (ATP5F1C, ATP6VID, ATP5F1A) in control (C1-C5) and irradiated groups (12Gy1-12Gy5). Bands marked in dashed square were chosen to represent the data.

Figure S7

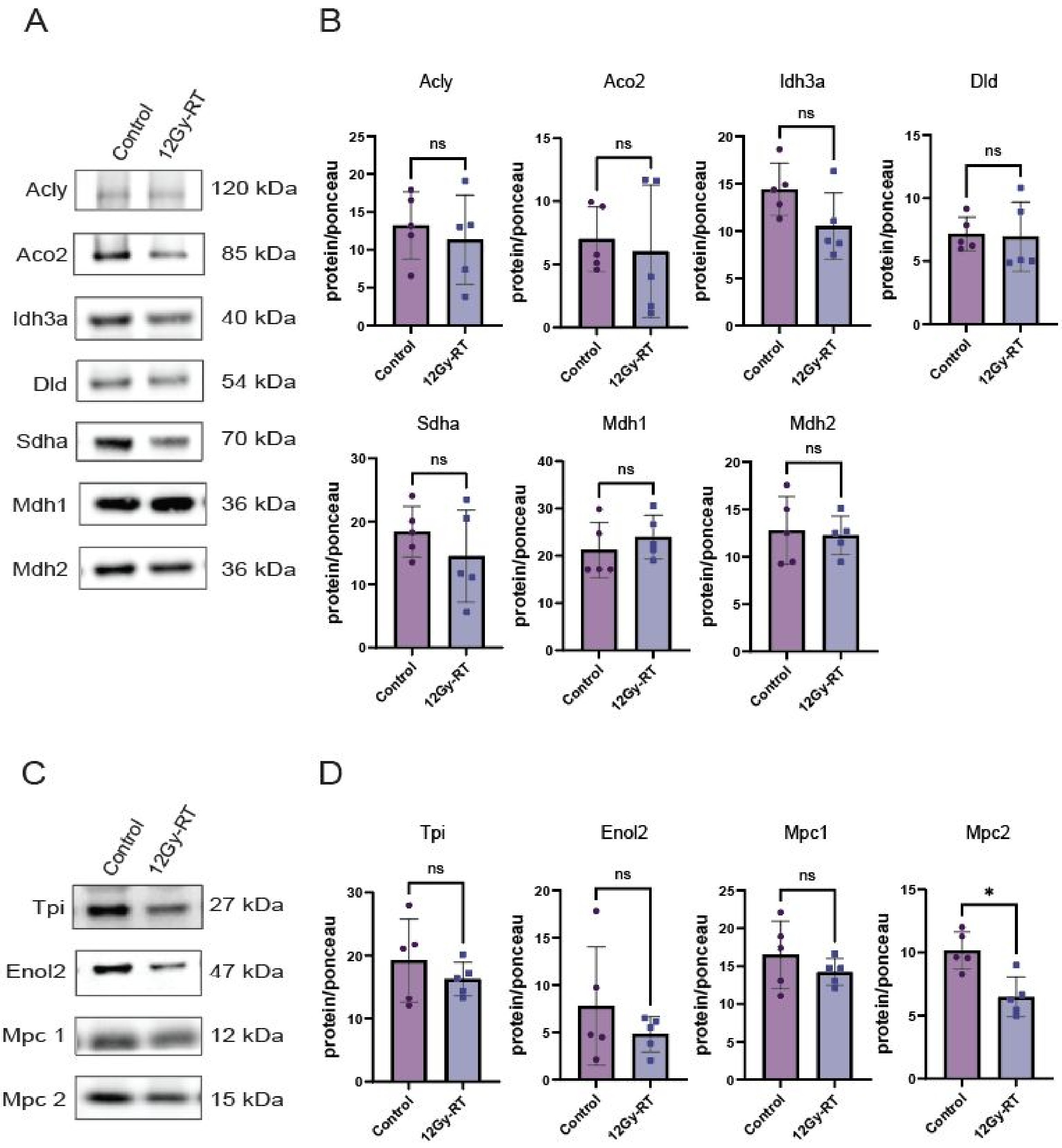

Figure S7: Immunoblot Validation of Protein Levels in Control and Irradiated Groups. (A) Representative immunoblots for TCA cycle proteins (ACLY, ACO2, IDH3A, DLD, SDHA, MDH1, MDH2) in control and irradiated groups. **(B)** Quantification of protein levels normalized to Ponceau-stained membrane (loading control). Analyses by Mann-Whitney test. **(C)** Representative immunoblots for glycolysis and pyruvate transport proteins (TPI, ENOL2, MPC1 and MPC2). **(D)** Quantification of protein levels normalized to Ponceau-stained membrane (loading control). Analyses by Mann-Whitney test.

Figure S8

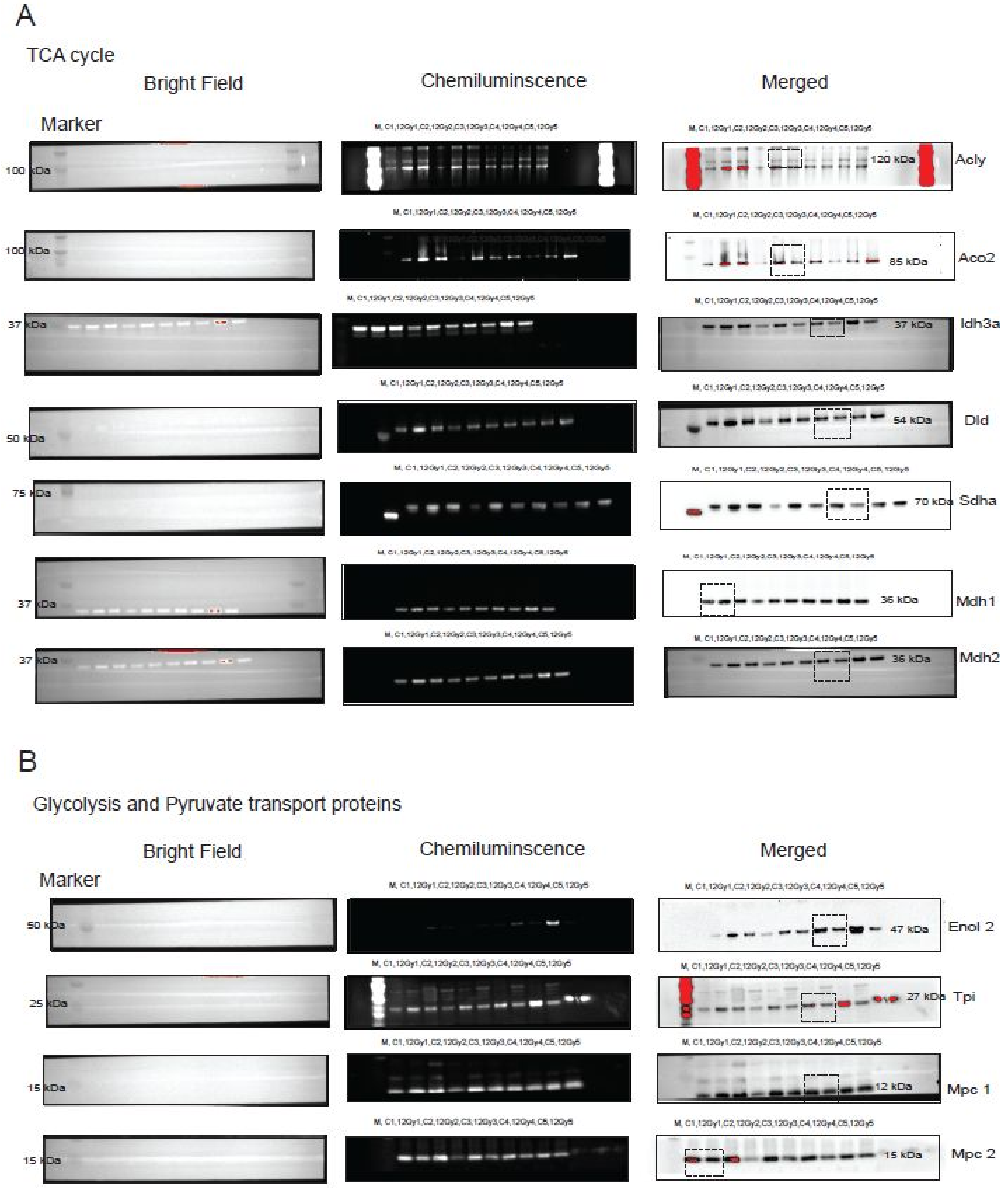
A
TCA cycle
Bright Field
Chemiluminscence
Merged
Marker
M, C1,12Gy1,C2,12Gy2,C3,12Gy3,C4,12Gy4,C5,12Gy5
100 kDa
120 kDa
Acly
85 kDa
Aco2
37 kDa
Idh3a
50 kDa
54 kDa
Dld
75 kDa
70 kDa
Sdha
36 kDa
Mdh1
Mdh2
B
Glycolysis and Pyruvate transport proteins
50 kDa
47 kDa
Enol 2
25 kDa
27 kDa
Tpi
15 kDa
12 kDa
Mpc 1
Mpc 2

Figure S8: Uncropped Immunoblots of Figure S7. (A) Uncropped immunoblots for TCA cycle proteins (ACLY, ACO2, IDH3A, DLD, SDHA, MDH1, MDH2) in control and irradiated groups. **(B)** Uncropped immunoblots for glycolysis and pyruvate transport proteins (TPI, ENOL2, MPC1 and MPC2) in control and irradiated groups. Bands marked in dashed square were chosen to represent the data.
